# Energy Based Vessel Sealing Devices in Thyroid Surgery: A Systematic Review to Clarify the Relationship with Recurrent Laryngeal Nerve Injuries

**DOI:** 10.3390/medicina56120651

**Published:** 2020-11-27

**Authors:** Mario Pacilli, Nicola Tartaglia, Alberto Gerundo, Giovanna Pavone, Alberto Fersini, Antonio Ambrosi

**Affiliations:** Department of Medical and Surgical Sciences, University of Foggia, Luigi Pinto Street, No. 1, 71122 Foggia, Italy; mario_pacilli.524126@unifg.it (M.P.); alberto_gerundo.534435@unifg.it (A.G.); giovanna_pavone.559403@unifg.it (G.P.); alberto.fersini@unifg.it (A.F.); antonio.ambrosi@unifg.it (A.A.)

**Keywords:** RNL injury, energy based vessel sealing devices, thyroid surgery

## Abstract

*Background and objectives:* The principal complications associated with thyroid surgery consist in postoperative recurrent laryngeal nerve (RLN) palsy, hypoparathyroidism, intra-operative and post-operative hemorrhage. In this paper, structured as a literature review, we describe the current knowledge and the technical improvements currently employed in the field of thyroid surgery, focusing on the contribution of energy based devices in relation with the reduction of the operating time and the odds of possible complication. *Materials and methods:* a relevant systematic literature search on Pubmed was carried out including works from 2004 through 2019, selecting studies providing information on the energy based devices employed in surgeries and statistic data concerning RNL (transient and permanent) injury and operative time. *Results:* Nineteen studies were reviewed, dealing with 4468 patients in total. The operative variables considered in this study are: employed device, number of patients, pathological conditions affecting the patients, surgical treatment, RNL injury percentage and the operating time, offering an insight on different patient conditions and their relative operative outcomes. A total of 1843 patients, accounting to the 41.2% of the total pool, underwent the traditional technique operation, while 2605 patients (58.3%) were treated employing the energy based devices techniques. Thyroidectomy performed by approaches different from traditional (for example robotic, MIVAT (Mini Invasive Video Assisted thyroidectomy)) were excluded from this study. *Conclusions:* The energy-based vessel sealing devices in study, represent a safe and efficient alternative to the traditional clamp-and-tie hand technique in the thyroidal surgery scenario, granting a reduction in operating time while not increasing RNL injury rates. According to this information, a preference for energy based devices techniques might be expressed, furthermore, a progressively higher usage rate for these devices is expected in the near future.

## 1. Introduction

Thyroidectomy is one of the most commonly performed operative procedures in neck surgery and it is the primary treatment option in the majority of thyroidal pathologies. Complication rates from total thyroidectomy are low: nowadays, the mortality rate for this procedure is approximately 0%, and the overall complication rate is less than 3% [1].

Total thyroidectomy is featured by a higher percentage of risk if compared with thyroid lobectomy, with a recent meta-analysis suggesting a pooled relative risk (RR) significantly greater for all complications, including recurrent laryngeal nerve injury (transient RR  =  1.7, permanent RR  =  1.9), hypocalcemia (transient RR  =  10.7, permanent RR  =  3.2), and hemorrhage/hematoma (RR  =  2.6) [2]. Furthermore, in total thyroidectomy subsists a rare but potential risk of bilateral recurrent laryngeal nerve injury, requiring tracheostomy. Surgeon experience most likely affects the risks of complications in thyroidectomy, as “de facto” demonstrated by higher volume surgeons, generally experiencing lower complication rates [3,4].

### General Complications in Thyroid Surgery

Hemorrhage is a rather uncommon but potentially serious complication in thyroid surgery, occurring in 0.3% to 4.2% of cases [5,6]. Intraoperative bleeding involves important structures and increases ripple effects of other anatomic complications. Usually patients present neck swelling, neck pain, and signs and symptoms of airway obstruction (dyspnea, stridor, hypoxia) in the worst scenarios. Therefore, if aspiration drains are not sufficient to prevent airway obstruction, bringing the patient back to the operating room might be the only competent choice.

Parathyroid hormone (PTH) and calcium levels are often measured after thyroid surgery, as hypocalcemia, secondary to postoperative hypoparathyroidism, is one of the most frequent (ranging between 7% to 37% [7,8]) complications in patients undergoing bilateral thyroid resection. Part of this variability is related to the variety of methods applied to define this complication [9]. The majority of patients with parathyroid dysfunction after thyroidectomy return to their normal function within a few weeks, depending on the extent of the gland’s devascularization or accidental resection of the parathyroid glands [10,11].

Tracheal injury associated with thyroidectomy was expressed less than 1% of the patients and is generally considered as a technical event during surgery, leading to the necessity of specialized treatment. Tracheal perforation, if encountered, requires management in centers of expertise with elevated volumes of thyroidectomies [12].

Recurrent laryngeal nerve (RLN) injury is a severe complication of thyroid surgery, accounting for a significant percentage of medico-legal claims. Nowadays the incidence of permanent RLN injury is sporadic, reaching 0.6–1.6% of total procedures; transient RLN injury is actually more frequent, with an incidence of 2–11% [13,14,15]. Several thyroidal dysfunctions account for a higher risk of RLN injury: notable differences are evident in case of surgery for toxic or substernal goiter, malignant tumors, and positive anamnesis for neck operations [16,17].

Dionigi et al. claim that the technical causes of RLN injury are transection, clamping, ligatures, suction, traction, thermal injuries and physical compression [18]. Other conditions linked to a higher risk of injury are: anatomic variability [19,20,21], malignancy [22], re-intervention [23,24] and the extent of the resection [25]. The visual identification of the RLN during surgery may reduce the incidence of injuries [22,26], moreover, the tubercle of Zuckerkandl and the parathyroid glands are landmarks that surgeons might exploit in order to identify the RLN. Some authors suggest use of microsurgical technique and loupes magnification as a safety and effective procedures, which can help surgical execution and avoid obstacles in thyroid surgery [27,28].

Unilateral vocal fold paralysis may be expressed with postoperative hoarseness while bilateral vocal-fold paralysis, typically occurring after total thyroidectomy, manifests itself determining a partial airway obstruction and biphasic stridor after extubating.

During surgical history, numerous technical advances concerning hemostasis have emerged. Energy based hemostatic devices are increasingly being employed and have proved to be potentially helpful in neck surgery. Not all of these “energy devices” rely on the same form of energy. LigaSure™ (LSJ Medtronic, Covidien product, Minneapolis, MN, USA) employs radio frequency [29], Harmonic Scalpel (Harmonic Focus; Ethicon, Johnson and Johnson, Cincinnati, OH, USA) instead, converts electric energy into mechanical vibration, producing ultrasonic movements [30]. Finally, Thunderbeat by Olympus, is a hybrid system that combines harmonic and radio frequency technologies, allowing a shorter period of application on the tissues [31].

Starting from the aforementioned information on the topic, this review is meant to inspect the role of energy based devices in the field of thyroid surgery, evaluating their pros and eventual cons and granting special considerations concerning several operative outcomes. This review gathered a large population of patients affected by different pathological conditions, giving the opportunity to inspect the state of art and the future perspective of energy based vessel sealing devices, shedding light on the following questions: do energy-based device reduce operative time (considering the different approaches to thyroidectomy) in comparison with the classic suture ligation technique? Are post-operative RNL injury rates (whether if transient or permanent) comparable with the traditional vessel sealing approach? Is there a preferable energy based vessel sealing device in terms of operative time and RNL injury rates? Do these outcomes vary in case different surgical techniques (total thyroidectomy, hemithyroidectomy, etc.) are employed?

The clinical history of 4468 patients, affected by benign and malignant pathologies (details provided in Table 1 and Table 2) was considered, as they were scheduled for surgery and post-operative outcome evaluations. The programmed interventions reviewed in this study consisted in total thyroidectomies, hemithyroidectomies and subtotal thyroidectomies, excluding minimally invasive techniques and re-interventions. From a technical standpoint the aforementioned operations relied on the employment of energy based vessel sealing devices, or on the traditional suture ligation technique. The comparison between these techniques was often inspected in the cited articles, but our effort focused on gathering a large amount of information regarding the post-operative outcomes, studying the eventual differences between the studies. The outcomes of interest in this work were the operative time and the RNL injury rates. Finally, our work gathered several study categories, such as prospective comparative studies, non-randomized retrospective reviews, randomized controlled trials, retrospective analysis, prospective randomized trials, randomized single center studies, prospective randomized studies and multicenter randomized controlled clinical trial.

## 2. Materials and Methods

An extensive search for relevant literature was carried out using MEDLINE (PubMed) collecting works published from 2004 to 2019. The keywords used for the search were: Ligasure™, Harmonic Scalpel, Thunderbeat, nerve injury, vessel sealing system, energy devices used with the Boolean operator ‘AND’ thyroidectomy.

Articles were selected considering the coherence with the topics addressed in our review, the availability of data concerning, the employed energy devices, the patients’ pathological conditions and the operative outcomes. The operative variables considered in this study are the following: adopted device, number of patients, pathological conditions affecting the patients, surgical treatment, RNL injury percentage and operating time. Data and information were extracted independently from the full text articles, according to the criteria enlightened in this section. Studies providing data on large number of patients and longer follow up intervals were preferred.

Thyroidectomies performed by approaches different from traditional (for example robotic, MIVAT) were excluded from this study. A language restriction to English and Italian was held, and two studies were not involved in our review. Several researches have been carried out on the use of Thunderbeat in thyroid surgery, although this tool is not as widespread as the previously mentioned devices. Studies on piglets demonstrated that this instrument can be wielded safely as close as 3 mm from RLN, on the condition to be actively used for a maximum time of 8 s [49]. Twelve studies were excluded because several data of interest were unavailable, three other papers showed overlapping information.

A total of nineteen studies, dealing with 4468 total patients were finally reviewed.

Recent studies comparing Ligasure and suture-ligation techniques evidenced nearly equivalent complication rates and hospital stay durations. The main differences were expressed in shorter operating time in patient belonging to the Ligasure group [33,35,36,50,51,52]. In other studies, the use of the Ligasure vessel sealer emerged as a safe alternative to the suture-ligation technique, even concealing a reduction in major complications rates [51,52,53]. No significant differences in outcomes, but higher operative costs were inspected by an Italian study [34].

A study conducted in 2006 found benefits from the use of the Harmonic device in comparison with the suture-ligation technique, citing a reduction of post-operative pain, blood loss and hypocalcemia, while no patients experienced nerve injury or permanent hypocalcemia in both groups. Moreover, mean operative times were slightly shorter within the Harmonic technique group [39]. Similar results in term of post-operative complications were found by other studies, which, in addition, referred a significant reduction in operating time [40,41]. A 2009 study compared suture-ligation, Ligasure and Harmonic techniques, finding the latter as the most efficient in terms of duration and costs, while the frequency of complications were substantially comparable [36]. The use of CFTP (Collagen Fibrinogen Thrombin Patch) showed a statistically significant reduction in drainage volume, and appears to potentially reduce bleeding complications [54,55].

The aim of our work is to elucidate the relationship elapsing between energy based vessel sealing devices and recurrent laryngeal nerve injuries by making a literature review, clarifying if a procedure or a selection of device might be preferred according to its operative and postoperative outcomes.

## 3. Results

The studies held by Petrakis, Cipolla, Manouras, Marrazzo et al. focused on the comparison between traditional vessel sealing technique and the Ligasure approach. In all of them was found a reduction in the operating time parameter, while the nerve injury odds were found to be equal or reduced with the employment of the Ligasure device.

Kirdak, Lepner et al. compared the outcomes of different surgical operations, dealing with total thyroidectomies, hemithyroidectomies and subtotal thyroidectomies. The Ligasure technique allowed net reduction of the operating time and percentages of nervous injury in the Kirdak et al. study. In opposition with the emerging trends, instead, the study held by Lepner et al. stated an increase in both operating time and nerve injury odds using the Ligasure device.

Pons et al. compared three operating techniques: the traditional, Ligasure and Harmonic device techniques. A substantial decrease in operating time was confirmed with the employment of the cited energy based devices, while the relatively limited statistic sample determined comparable values concerning the nervous injury percentages.

Miccoli, Yildirim, Lombardi et al. published works dealing with the comparison between the hand and knot technique with the Harmonic device technique. In the totality of these studies a reduction in the operating time parameter was identified. Miccoli et al. did not record any nervous injury, Yildirim et al. identified a notable reduction of RNL injury employing the Harmonic device, while Lombardi et al. stated a higher risk with the aforementioned tool.

Back et al. studied the outcomes of three different instrument: the Harmonic device, the Ligasure device and the Thunderbeat device, identifying no nervous injury with the first tool, but progressively higher rates employing the Ligasure and the Thunderbeat device approaches. The results involving the operating time were found to be miscellaneous, not highlighting a faster approach, but rather comparable surgeries from a timing standpoint.

Shemen et al. focused their work on the sole Harmonic device, with similar results in terms of operating time if compared to studies employing with the same approach. No RNL injury was recorded.

Chang et al. proposed a study investigating a wider comparison between the traditional and the sutureless technique, showing close results for what concerns the nervous injury rates, but a beneficial effect of energy based devices on the operating time.

Coiro, Bircan, Kuboki, Hirunwiwatkul et al. furtherly compared the traditional and Ligasure approaches. The first three found the energy based device approach to be more effective in terms of operating time reduction, yet determining higher rates of nervous injury. The latter, instead, showed comparable RNL injury rates between the two techniques, still considering the Ligasure as the least time-consuming approach.

Hwang et al. compared two sutureless approaches: Ligasure and the Harmonic ones. The first led to slightly better results in both of the outcomes studied in this work.

Finally, Al Dhairy et al. studied the different outcomes led by the employment of the traditional, the Harmonic and the maxium approaches. The first one was found to be the least favorable option, burdened by the highest rates of nervous injury and the longest operating time. The Harmonic and the maxium approaches determined comparable operating time durations, but the latter caused a higher number of nervous injuries.

## 4. Discussion

The thyroid gland is featured by a complex and developed vascularization, therefore the correct recognition of vascular and nervous structure, nonetheless the careful dissection of the aforementioned are paramount for success in this field of surgery. The historically native clamp-and-tie ligation technique has been recently flanked by energy-based devices approaches and the constantly increasing employment of these tools in thyroid surgery allowed a significant reduction in main operating time in most of the available studies in literature. The Ligasure radio frequency device, the Harmonic scalpel, employing ultrasonic movements and the Thunderbeat hybrid system, progressively allowed the compression of the notably frequent and time consuming vessel sealing stages in thyroidal surgery. These devices, originally designed for other fields of surgery (such as abdominal surgery, for instance) proved to be reliable in the context of thyroid surgery, granting safety, yet improving several operative and post-operative outcomes.

LigaSure™ (LSJ Medtronic, Covidien product, Minneapolis, MN, USA) employs radio frequency, a signal that can seal vessels up to 7 mm in maximum diameter [29]. The tip of the ultrasonic instrument Harmonic scalpel, which is not protected as in radio frequency instruments, reaches the maximum temperature fairly quickly, therefore might be responsible for tracheal and major vessel injuries, but it is able to seal vessels up to 5 mm in diameter [30].

The main focuses of this study were the variables of mean operating time of the surgery and the percentages of post-operative RNL injury. Accurate knowledge of anatomy and pathophysiology, complications incidence and pathogenesis, joint with a careful surgical performance are essential to grant the fewest possible complications. Most of the reviewed studies stated a reduction in operating time suggesting a preference for energy based devices techniques to the traditional one. Moreover, these recent approaches granted statistically comparable rates of RNL injuries [32,33,34,35,36,37,38,39,40,41,42,43,44,47,48,56,57]. Several studies demonstrated a better performance of the Harmonic scalpel in comparison to the Ligasure device in regard of the operative timing reduction [35,39,44], while one study found its employment detrimental to the decrease of the aforementioned variable [46]. The strong points of this study consist in a rather large number of patients considered, mostly involved in comparative evaluations between different energy devices and traditional hand and tie technique.

The limitations of this study consist in several studies not mentioning [38,41,57] the specific pathological conditions affecting the patients prior to the interventions, lowering the richness of other parameters of interest. One study did not specify the employed devices in their “sutureless” technique interventions, reducing the precision of the eventual single devices’ comparison [43]. In our opinion these limitations did not actually decrease the quality of the main questioned outcomes, providing instead a large number of patients’ clinical history data to the cause. Most of the studies cited, nonetheless, provide these information, consenting the appreciation of the outcomes of the aforementioned surgeries, adding a wide collection of data and granting a comprehensive outlook on the recent history and on the state of the art in thyroidal surgery.

## 5. Conclusions

The three aforementioned devices, according to the scientific literature, represent a safe and efficient alternative to the traditional clamp-and-tie hand technique in thyroidal surgery scenario, granting a reduction in operating time while not increasing complication rates. In this study we focused on operative time durations and RNL injury rates. The operative times were typically reduced with the employment of energy based devices, while the RNL injury rates expressed numbers and ratios statistically indifferent to the sealing device employed in single articles scenarios. Accordingly to our data, we believe that the experience and the skills of the surgeon play a decisive role, more than the specific energy based devices employed, in thyroid surgery.

## Figures and Tables

**Table 1:** The main operative variables examined in the study. (T: traditional, L: Ligasure™, H: Harmonic, Tb: Thunderbeat, Mx: maximum. TT: total thyroidectomy HT: hemithyroidectomy ST: subtotal thyroidectomy).

Study	Devices	Cases	Treatment	Nerve Injury *	Op. Time (min)
Petrakis et al., 2004 [32]	T vs. L	517 Total	517 TT		
		247 Traditional		2.00%	86.0 ± 22.0
		270 Ligasure		0.40%	71.0 ± 14.0
Kirdak et al., 2005 [33]	T vs. L	58 Total			
		28 Traditional	10 HT	10.70%	99.8 ± 12.53
			9 TT		128.98 ± 19.73
			9 ST		117.33 ± 11.77
		30 Ligasure	8 HT	3.30%	77.38 ± 13.71
			8 TT		102.5 ± 16.69
			14 ST		103.36 ± 23.48
Cipolla et al., 2008 [34]	T vs. L	105 Total	105 TT		
		52 Traditional		1.92%	110.0 ± 15.6
		53 Ligasure		1.88%	104.0 ±12.7
Pons et al., 2009 [35]	T vs. L vs. H	60 Total	60 TT		
		20 Traditional		5.00%	151.0 ± 15.0
		20 Ligasure		0.00%	122.0 ± 10.0
		20 Harmonic		5.00%	114.0 ± 9.0
Lepner et al., 2007 [36]	T vs. L	403 Total			
		199 Traditional	10 HT	0.50%	54.0 ± 27.2
			9 TT		78.3 ± 34.4
			9 ST		60.4 ± 19.2
		204 Ligasure	8 HT	1.00%	75.3 ± 20.6
			8 TT		104.8 ± 28.5
			14 ST		106.0 ± 37.7
Manouras et al., 2008 [37]	T vs. L	184 Total	184 TT		
		90 Traditional		0.00%	101.6 ± 3.6
		94 Ligasure		0.00%	87.3 ± 2.2
Marrazzo et al., 2007 [38]	T vs. L	50 Total	50 TT		
		25 Traditional		4.00%	92.4 ± 27.5
				4.00% **	
		25 Ligasure		4.00%	60.0 ± 14.8
Miccoli et al., 2006 [39]	H vs. T	100 Total	100 TT		
		50 Traditional		0.00%	46.7 ± 10.8
		50 Harmonic		0.00%	40.0 ± 6.8
Shemen et al., 2002 [40]	H	105 Total			
		105 Harmonic	50 HT	0.00%	99.1
			55 TT	0.00%	134.9
Yildirim et al., 2008 [41]	H vs. T	104 Total	104 TT		
		54 Traditional		9.00%	105.0 ± 16.0
				1.85% **	
		50 Harmonic		2.00%	77.9 ± 12.5
Lombardi et al., 2008 [42]	H vs. T	200 Total	100 TT		
		100 Traditional		1.00%	75.2 ± 23.5
		100 Harmonic		2.00%	53.1 ± 20.7
Chang et al., 2011 [43]	H e L vs. T	1935 Total	1935 TT		
		772 Traditional		2.10%	75.2 ± 23.5
				0.3% **	
		1163 “Sutureless”		2.00%	53.1 ± 20.7
				0.5% **	
Back et al., 2019 [44]	H vs. L vs. Tb	75 Total			
		25 Harmonic	18 HT	0.00%	18 ± 3,53
			7TT		
		25 Ligasure	15 HT	4.00%	19.42 ± 4.77
			10 TT		
		25 Thunderbeat	15 HT	8.00%	16 ± 3.39
		32 Ligasure		12.5%	58.9 ± 23.6
Hirunwiwatkul et al.2013 [45]	L vs. T	40 Total	40 HT		
		20 Traditional		NM	83.3 ± 16.1
				5.00% **	
		20 Ligasure		NM	62.4 ± 15.9
				5.00% **	
Hwang et al., 2014 [46]	L vs. H	126 Total			
		64 Ligasure	39 TT 25 HT	1.56%	104.3 ± 3
				1.56% **	
		62 Harmonic	39 TT 23 HT	1.61%	106.6 ± 2.1
Kuboki et al., 2014 [47]	L vs. T	82 Total			
		43 Traditional	15 TT 28 HT	12.8%	104.2 ± 41.4
				2.6% **	
		39 Ligasure	14 TT 25 HT	16.3	89.2 ± 42.8
Al Dhairy et al., 2016 [48]	L vs. T vs. Mx	80 Total	80 TT		
		26 Traditional		11.5%	113 ± 10.9
				11.5% **	
		26 Harmonic		3.84%	93 ± 13
		28 Maxium		7.14%	92 ± 10.6
				3.57% **	

* Transient injury, ** permanent injury.

**Table 2:** The pathological conditions affecting the patients involved in the study. (L: Ligasure™, H: Harmonic, T: traditional, Tb: Thunderbeat, Mx: maxium, MNG: multi nodular goiter. TMN: toxic multi nodular goiter, GD: Grave’s disease. AIT: autoimmune thyroiditis, ADE: adenoma. MAL: malignancy, M + M: multinodular goiter + malignancy, HYP: hyperplasia. UNDN: undetermined nature nodule, OTH: other, BEN: benign disease).

Study	DEVICE	MNG	TMN	GD	AIT	ADE	MAL	M + M	HYP	UNDN	OTH	BEN
Petrakis et al., 2004 [32]	L	270										
	T	247										
Kirdak et al., 2005 [33]	L	27		1			2					
	T	22		3			3					
Cipolla et al., 2005 [34]	L	33	4	3	8		5					
	T	31	5	2	8		6					
Pons et al., 2009 [35]	L	20										
	H	20										
	T	20										
Lepner et al., 2007 [36]	L	165		17			22					
	T	169		18			12					
Manouras et al., 2008 [37]	L	6			52	10	8	12	2		4	
	T	52			8	4	10	4	4		8	
Marrazzo et al., 2007 [38]	L	NM	NM	NM	NM	NM	NM	NM	NM	NM	NM	NM
	T	NM	NM	NM	NM	NM	NM	NM	NM	NM	NM	NM
Miccoli et al., 2006 [39]	H	37	3	7			3					
	T	38	3	6			3					
Shemen et al., 2002 [40]	H						41					64
Yildirim et al., 2008 [41]	H	NM	NM	NM	NM	NM	NM	NM	NM	NM	NM	NM
	T	NM	NM	NM	NM	NM	NM	NM	NM	NM	NM	NM
Lombardi et al., 2008 [42]	H	46	12	6			8			28		
	T	48	16	6			9			21		
Chang et al., 2011 [43]	L + H	641		131	92	54	199				46	
	T	453		73	44	40	125				37	
Back et al., 2019 [44]	H						24					1
	L						20					5
	Tb						22					3
Coiro et al., 2015 [45]	L + T	154		8	17				11			
Bircan et al., 2014 [46]	L	NM	NM	NM	NM	NM	NM	NM	NM	NM	NM	NM
	T	NM	NM	NM	NM	NM	NM	NM	NM	NM	NM	NM
Hirunwiwatkul et al.2013 [45]	L									20		
	T									20		
Hwang et al., 2014 [46]	L						64					
	H						62					
Kuboki et al., 2014 [47]	L			10		6	22				1	
	T			10	1	12	19				1	
Al Dhairy et al., 2016 [48]	H	25					1					
	Mx	25					2					
	T	26					1					
